# Preventive Dentistry

**Published:** 1917-04

**Authors:** Russel W. Bunting

**Affiliations:** Ann Arbor, Mich.


### PREVENTIVE DENTISTRY

BY RUSSEL W. BUNTING, D.D.S., ANN ARBOR, MICH.

Some three years ago we were told by a prominent member of the medical profession that the further advancement of the science of preventive medicine depended upon our ability to eliminate oral infections which might be harmful to the health of the whole economy. The efforts of medical investigation and practice have been directed largely in late years toward the prevention of disease, with the result that the death rate has been decreased, living has been made more comfortable, and years have been added to the span of life. And now we, as dentists, are told that further progress along these lines is dependent upon our ability to prevent the entrance of infection to the body through the avenue of the mouth.

We have heard and responded to the call. We have made wholesale examinations of our patients by the X-ray and other methods of diagnosis to determine to what extent oral infections exist. We have been astonished by the information which we have gained, and by the frequency with which sepsis occurs even under conditions which had formerly seemed to us to be most favorable. Moreover, certain members of our profession, by study and experimentation, have demonstrated that these oral infections have the property, in some instances, of producing lesions and metabolic disturbances of tissues and organs of the body far remote from the original seat of infection. The result has been the awakening of a keen interest in oral infections among dentists, the medical profession, and the laity.

In a consideration of the entrance of infections of various orders to the tissues of the body, we know that nature has built up a strong and efficient barrier against such organisms. Disease-producing bacteria exist all about us, and would annihilate the whole race in a short space of time were it not for the defenses of the individual against the entrance and propagation of such bacteria in the tissues. The skin and mucous membrane are the external defenses, and when intact and in health there are but few organisms which can penetrate them. In the mouth, therefore, pathogenic and nonpathogenic bacteria may have their habitation in large numbers and be entirely harmless to the body, as long as the mucous membrane of the mouth remains intact. It is when a lesion in this protective covering has occurred that infection may enter the oral tissues to produce local or general effects.

### AVENUES OF ENTRANCE OF INFECTION TO THE SYSTEM

The most vulnerable spot in the mucous membrane covering of the mouth is to be found in the gingival tissues, the openings through which the teeth are pushed in eruption, and which exist as long as the teeth remain in position. It is in these places that the integrity of the protective covering is most easily and frequently broken, and through which infections may most readily gain entrance to the deeper tissues. In addition to the lesions of the mucous membrane, the integrity of the teeth is destroyed by dental caries offering a means of access for infectious organisms through the cavity in the tooth and the root canal to the tissues of the periapical space. Through these two avenues the great majority of oral infections enter the tissues to produce their specific results. These are the gateways of infection which lie within our domain, for the closure of which preventive medicine has asked our cooperation.

Dentistry is everywhere rising today to meet the demand. Every effort is being turned toward the eradication of periapical and peridental infections. Methods of diagnosis and treatment of dental abscess and pyorrhetic conditions are being perfected whereby serious oral infections are being eliminated and the tissues restored to normality—all of which has been of great service to medical practice in the treatment of many general disease conditions of an infectious nature. The demand for such service is universal, and the amount of oral disease to be remedied is almost appalling, so that dentistry will be very busy during the next few years in diagnosing and eliminating the untoward conditions which exist in the mouths of a large percentage of the people. But praiseworthy as this form of treatment may be, it falls short of the ideal service, in that it often fails to repair the damage which has been done, or to entirely eliminate the harmful foci of infection. The ideal dental service is that by which the lesions through which the infections originally enter will be prevented, and the normal protection of the body be conserved. Could dentistry prevent the occurrence of dental caries through means by which the teeth would remain intact, and peridental or gingival infections, by which the normal resistance of mucous tissues would be conserved, the mouth would be most effectively eliminated as a source of general infection. It is, then, the duty of Dentistry, if she wishes to fill her place in the great work of preventive medicine, to turn her attention and to exert her best efforts toward the control and eradication of these two dental diseases.

### SUSCEPTIBILITY AND IMMUNITY TO DENTAL DISEASE

In a consideration of the problem of prevention of dental lesions we note that a certain small percentage of people go through life seemingly immune to dental disease, and that certain others are but slightly susceptible, in distinct contrast to the majority, who frequently suffer from one or both forms of dental disease. Two questions suggest themselves for solution, namely—

(1) What oral conditions determine immunity to dental disease?

(2) May immune conditions be produced artificially in mouths which are naturally susceptible?

In regard to the first question, we observe that a general bodily health and a well-developed physique usually imply that the tissues of the mouth are well formed, well nourished, and resistant to disease. We also note that certain environmental states exist in immune mouths which preclude their injury from infective or irritating materials. For instance, the lactic acid organisms of caries exist and produce their characteristic fermentations in the mouths of immunes as well as in those of susceptibles, but physical or physicochemical conditions prevail in the mouths of the former which prevent the decalcification of the teeth and the inception of caries. These determinal conditions which control the occurrence of dental caries have been discussed theoretically in many contributions to that study, and can not well be considered in a treatise of this nature. One general feature is very evident: that in all types of mouths those surfaces which are continually cleansed by the force of mastication are usually immune to caries. Such surfaces are frequently rubbed by the excursions of food across them, and other forces of mastication also interfere with the localization of caries bacteria, whereby their characteristic action is prevented. Therefore it may be stated that, as a rule, the more self-cleansing the teeth are, the less susceptible are they to caries. This self-cleansing property is dependent upon the anatomical form and arrangement of the teeth, the smoothness of their surfaces, the character of food—hard or soft—which is eaten, the thoroughness with which mastication is performed, and the viscosity and quantity of the saliva. In the light of our present knowledge, these are evident and potential factors in the production of dental caries.

Pyorrhea and peridental diseases almost invariably begin as inflammation of the gingival tissues. These inflammations may be the result of trauma, such as impaction of food during mastication, accumulations of calculi on the teeth, or malocclusion. It may also be produced by chemical irritants such as drugs or the products of fermentation and other bacterial action. The effect of such local irritations may be enhanced by or combined with disturbances of circulation due to systemic causes. In case of immunity to peridental disease we see that one of two conditions obtains—namely, either the gingivae are not subjected to harmful or irritating influences, or the character of the circulation is so good that the health of those tissues is maintained in spite of the untoward conditions.

Natural immunity to dental disease is therefore largely dependent upon the full normal health and physiological function of the dental tissues combined with the absence of extraneous substances capable of producing injury. In answer, then, to our second question, regarding the possibility of artificially producing immunity to dental disease, we may say that it is most nearly approximated by those measures which assist the mouth to perform its full normal function and tend to continuously rid the oral tissues of harmful or irritating agencies.

### MEANS OF ASSISTING NATURE IN THE PREVENTION OF DENTAL DISEASE

What, then, are the means by which we may assist nature in the prevention of dental disease? According to our present knowledge such assistance may be classified in three groups:

(1) Those measures by which the teeth may attain their full development and be brought into normal relation with each other.

(2) The restoration of anatomical forms in teeth which have been mutilated by disease, and the replacement of teeth which have been lost.

(3) The cleansing of the teeth of all extraneous material, and teaching the patient to give intelligent mechanical assistance in maintaining oral hygiene.

Theoretically, there is reason to believe that the application of such measures would result in the reduction of dental disease of both orders, and this has practically proved to be true. For many years there have been certain members of our profession who have devoted themselves largely to the practice of oral prophylaxis, intensively directing their energies to these three general lines of procedure. The reports of these operators and examination of their cases show an unmistakable improvement in oral conditions. In a large number of cases peridental disease has been arrested, and in practically all cases its primary inception has been prevented. Dental caries is not so successfully prevented as are peridental diseases, but the progress of caries is usually impeded, and in a large percentage of cases the teeth seem to be practically free from attack. The validity of these statements has been corroborated by the author in observing the changes in mouth conditions which have occurred in over fifty patients who have been given personal treatment along these lines. These patients were suffering from one or both of the two common dental diseases, many of them being very seriously affected. The results of the application of preventive measures after two years of observation have been that practically all peridental disease has been arrested, and no new involvement is to be seen. But six cases of recurrence of caries have been found, although several of these patients were presenting that number each year previous to the inauguration of the preventive measures. These personal observations have convinced the writer that such oral hygienic and prophylactic measures when completely carried out *do* prevent dental disease in a large number of cases, and in every instance the resistance of the dental tissues is raised.

The fact that the practice of oral hygiene does not always prevent dental disease does not indicate that it is ineffective. The problems of preventive dentistry are not unlike those of preventive medicine. The latter are largely concerned with the control of infections which are the active cause of the large number of infectious diseases to which man is susceptible. If by some means preventive medicine could accomplish the complete annihilation of all disease-producing bacteria, the great majority of human ills would thereby be prevented, and the span of life would be lengthened by many years; but this, at the present time, is impossible. Medicine has therefore sought to make the most of its knowledge, incomplete though it may be, in seeking to reduce the susceptibility to infection in the individual and to increase bodily resistance against disease organisms. The results obtained in the prevention of smallpox, typhoid, diphtheria, tetanus, cholera, tuberculosis and many other infectious diseases are eloquent evidence of the efficacy of the measures employed, in spite of the fact that they are not equally effective in all cases.

So, also, in the practice of preventive dentistry by means of oral hygienic measures, it is not possible to remove all infection and all unfavorable influences which may exist in the mouth, but it is possible to reduce them, and at the same time to increase the health and resistance of the dental tissues. In this manner the balance between the strength of the attacking force and the resistance of the tissues is swung toward immunity and away from susceptibility. Within certain limits the thoroughness with which the prophylactic measures may be or are carried out will determine the degree of immunity attained. In some cases dental disease will be prevented, while in others it will be made less frequent of inception and less virulent in attack.

### ORAL HYGIENE AND PROPHYLAXIS THE ONLY PRACTICAL MEANS OF PREVENTION

The principles of oral hygiene and prophylaxis, when practiced in their broadest sense, are inhibitive of dental disease, and as they are at the present time the only known practical methods of prevention they may well be called the principles of preventive dentistry. But if these principles are to be of real value in preventive medicine, the practice of them must not be limited to the specialist who devotes his whole attention to one particular phase. True it is that those who give their whole time and thought to such practice may most effectively apply the principles involved, but the scope of such men is altogether too limited to reach the masses in dental practice and to make dental prevention the general good which it should be. It rests, then, with the general practitioner to apply these principles in the daily routine of his dental operations. He must learn to recognize the unfavorable influences in the mouth and to seek cooperation on the part of his patients in their removal. In this manner he will most effectually keep the mouths of his patients in health, and prevent a large part of dental disease.

### APPLICATION OF THE PRINCIPLES OF ORAL HYGIENE IN GENERAL PRACTICE

Let us consider briefly the application of the principles of oral hygiene to the general practice of dentistry. It is evident that the assistance which is to be given to the development and arrangement of the dental tissues can most effectively be applied during early childhood. Proper advice to parents as to the care of the mouth and teeth of their children has often a profound effect. It is also important to warn them against excessive use of soft and pappy foods in the diet of the child, teaching them that vigorous and thorough mastication of hard foods is a great stimulus to the proper growth of all dental tissues.

It is at this age that the most may be done for children in the way of orthodontic assistance, by which teeth in abnormal relations may be corrected and given the opportunity to properly functionate throughout the life of the individual—a factor of great importance in the prevention of dental disease. It is not always possible to obtain, nor may we expect, intelligent cooperation on the part of these little patients, so that in a large percentage of cases, especially where the forces of disease are severe, cavities appear in the teeth. Frequent examinations are therefore necessary to assist in the cleansing of the mouth, and to discover and fill the cavities when they are small. In this manner the teeth of the child may be preserved free from abscesses and in full function, to play their normal part in the physiological exchange of dentitions.

In this connection we shall be interested in the development of an institution in this section which aims to practice these principles of child hygiene and preventive dentistry upon a large scale. It is the purpose of the Eastman Dental Infirmary to direct all the work which they do for children toward the prevention of dental disease and the assistance of the dental tissues to attain their full normal development. The work of this institution during the next ten years will be a huge experiment in child hygiene and dental prevention, the results of which will be of inestimable value in judging the real efficiency of such preventive measures.

### IMPORTANCE OF RESTORATIVE OPERATIONS AS AN AID TO ORAL HYGIENE

In the treatment of patients who have passed the formative period somewhat different conditions prevail. Here we usually find lesions of dental tissues of one or both orders. Palliative and reparative treatment is therefore indicated. Fillings, crowns, bridges, plates, etc., are necessary to replace tissues lost by dental disease, but the important feature from a preventive standpoint is that these restorations shall restore the teeth to their full anatomical contour, and give normal occlusion and interproximal contact. High and definite marginal ridges on the molars and bicuspids, and proper mesiodistal contours, affording tight approximal contacts between all the teeth, have a most important function in the self-cleansing of the mouth. When operative measures are performed with these principles in mind the food is more effectually masticated and is directed away from the interproximal spaces rather than into them. Many practitioners perform all their operations with a view to restoring these anatomical relations in their own work, but overlook certain fillings with flat occlusal surfaces and overhanging gingival margins which have been doing a pernicious service for some time. Many such fillings may well be replaced, even though there is no recurrence of caries around them, and others may be reshaped and made to fairly well conform to the normal by a little judicious grinding and polishing. The "tin can" type of gold crown, with its ill-fitting and irritating edges, is a too frequent type of malefactor, in the presence of which preventive dentistry is impossible. So also are all the other forms of reparative dentistry which do not conform to the normal contour and relations of the teeth. It is gratifying to see that all the modern forms of operative and prosthetic procedures are in every way attempting to conform to the highest anatomical ideal, and practice of this character will most effectively assist in the self-cleansing of the mouth.

Another very important feature is the relief of undue and unnatural stress of one tooth upon another. We frequently see cases in which teeth come into occlusion by a sliding action, in which they and their antagonists are deflected laterally each time the teeth come together. The results in an undue strain upon the peridental tissues, which induces injury and infection. All of this may usually be remedied by the judicious use of stones and carbon paper.

### PROPHYLAXIS

With regard to the measures known as prophylaxis of dental tissues much has been said and written in the past few years which can not be given a full consideration at this time. There are, however, certain salient features which are of special importance and deserve to be mentioned. Every operator who is conscientious in his service to his patients attempts to remove all tartar and food materials from the surfaces of the teeth and gums of every patient, but this service, commendable as it may be, is not prophylaxis. It is more properly known as "cleaning teeth." True prophylaxis involves the preparation of the dental tissues so that they may be continuously kept relatively clean. The large majority of mouths as we see them in dental practice can not be properly cared for by the patient after a single cleaning, and deposits immemediately begin to form which tend to produce caries and peridental irritation. It is then necessary to supplement the cleaning measures by smoothing and polishing procedures that will render the surfaces of the teeth capable of being cleansed by the personal efforts of the patient. By the use of fine files or planing instruments the roughened tooth surfaces beneath dental calculi, and those gingival and approximal areas which have been roughened by fermentation or calculi formation, may be smoothed and given homogeneous surfaces. These and other roughened surfaces of the teeth and fillings may then be polished with fine abrasive and polishing powders, using the porte-polishers, Young's engine cups, and the wide dental tape. The effect of such prophylactic measures when carried out in a thorough manner and combined with other oral hygienic measures is that the gum tissues are relieved of irritation, the teeth are rid of caries-producing plaques, and continuous oral hygiene is made possible.

### IMPORTANCE OF THE PATIENT'S COOPERATION

In many instances the simple reorganization of the mouth along hygienic lines is sufficient to produce a permanent improvement in the conditions. But the great majority of mouths at their best are not self-cleansing, and need the daily assistance and cooperation of the individual to attain oral health; it should therefore be the rule that the enthusiastic cooperation of the patient be enlisted in a daily care of the mouth that is most rigorous and thorough. By the use of suitable dentrifices and brushes of a hard, stiff type, with a stroke beginning the circulation of soft tissues is stimulated and the teeth the circulation of soft tissues is stimulation and the teeth are cleansed of debris on both their lingual and labial surfaces. This must be supplemented by a cleansing of the approximal surfaces of the teeth, those most susceptible to caries, by the careful use of the wide dental floss. If this can be well accomplished once a day, and in addition a light brushing be given after each meal, a continuous state of mouth cleanliness will usually be maintained. With regard to the selection of dentifrices, certain mouths seem to need a preparation having a slight abrasive action, while others may be well cared for with those containing only the finest grades of calcium carbonate. We have published a short description of a number of the common varieties in the *Journal of the National Dental Association*, for August, 1915, to which those who desire this information are referred.

For the best results in this form of endeavor it is highly desirable that such patients be seen occasionally, some more frequently than others, for the purpose of assisting them in the care of their mouths and for the detection of dental diseases at their inception.

### THOROUGH MASTICATION AN IMPORTANT AID TO ORAL HYGIENE

It must also be remembered that one of the greatest aids to mouth cleanliness is the habit of thorough and vigorous mastication of hard foods, by which the teeth are mechanically scrubbed in a natural manner, a copious flow of saliva is induced by which the mouth is cleansed, and the gums and peridental tissues are massaged. This action may be intensified by the chewing of tough gums, such as spruce gum, directly after each meal.

To many general practitioners the procedures as outlined may seem complicated and impracticable of accomplishment in everyday dental service. This, indeed, might be true were they all adopted at once, but the object of the writer is to present an ideal which may be the *motif* of general practice to which all operations may be made to conform as nearly as possible. Nor is the ideal impossible, for we may point to a goodly number of general practitioners who for many years have been successfully putting these principles into effect in a large percentage of their cases. They have proved that preventive dentistry is possible and practicable in general practice. It only remains for the mass of dental practitioners to adopt these measures and this ideal to make dental prevention a reality. No one can truly appreciate the benefits to be derived from such methods of practice by hearing them described, or even by seeing the results of others. They can only be realized by personal application of such principles and by personal observation of the resultant changes in the oral conditions. Thus it is that those who doubt are convinced; and only those who will not see, disagree. One cardinal characteristic is demanded, and that is thoroughness. Halfway measures will not accomplish prevention, but the complete fulfilment of our present known methods of oral hygiene not only prevents dental disease but is of direct benefit to the body as a whole. Let us, then, rise to meet the situation; let us practice to the best of our ability the methods of prevention which we now know, and widen the scope of such service until it shall reach all people, to the end that we may most effectively perform our part in the great work of preventive medicine of today.—*Dental Cosmos* for March, 1917.

